# Regulation of Chemokines and Cytokines by Histone Deacetylases and an Update on Histone Decetylase Inhibitors in Human Diseases

**DOI:** 10.3390/ijms20051110

**Published:** 2019-03-05

**Authors:** Himavanth Reddy Gatla, Nethaji Muniraj, Prashanth Thevkar, Siddhartha Yavvari, Sahithi Sukhavasi, Monish Ram Makena

**Affiliations:** 1 Department of Pediatric Oncology, Johns Hopkins School of Medicine, Baltimore, MD 21287, USA; hgatla1@jhmi.edu; 2 Department of Oncology, Johns Hopkins School of Medicine, Baltimore, MD 21287, USA; nmunira1@jhmi.edu; 3 Department of Microbiology, New York University, New York, NY 10016, USA; prashanth98@gmail.com; 4 Department of Epidemiology, Johns Hopkins Bloomberg School of Public Health, Baltimore, MD 21205, USA; syavvar1@jhu.edu; 5 Center for Distance Learning, GITAM University, Visakhapatnam, AP 530045, India; Sahithisukhavasi1695@gmail.com; 6 Department of Physiology, Johns Hopkins School of Medicine, Baltimore, MD 21205, USA

**Keywords:** HDACs, chemokines, cytokines, HDAC inhibitors, human diseases

## Abstract

Histone acetyltransferases (HATs) and histone deacetylases (HDACs) counteract with each other to regulate gene expression by altering chromatin structure. Aberrant HDAC activity was reported in many human diseases including wide range of cancers, viral infections, cardiovascular complications, auto-immune diseases and kidney diseases. HDAC inhibitors are small molecules designed to block the malignant activity of HDACs. Chemokines and cytokines control inflammation, immunological and other key biological processes and are shown to be involved in various malignancies. Various HDACs and HDAC inhibitors were reported to regulate chemokines and cytokines. Even though HDAC inhibitors have remarkable anti-tumor activity in hematological cancers, they are not effective in treating many diseases and many patients relapse after treatment. However, the role of HDACs and cytokines in regulating these diseases still remain unclear. Therefore, understanding exact mechanisms and effector functions of HDACs are urgently needed to selectively inhibit them and to establish better a platform to combat various malignancies. In this review, we address regulation of chemokines and cytokines by HDACs and HDAC inhibitors and update on HDAC inhibitors in human diseases.

## 1. Introduction

### 1.1. Histone Deacetylases

Histone acetyltransferases (HATs) and histone deacetylases (HDACs) counteract with each other to regulate gene expression by altering chromatin structure [1,2]. HDACs remove acetyl groups on N-terminal extensions of the core histones, leading to a closed chromatin configuration and subsequently result in transcriptional repression [3]. More than eighteen different HDACs have been identified to date based on their phylogenetic analyses and sequence homologies with the yeast proteins. Class I HDACs (HDACs 1, 2, 3 and 8), class II HDACs (HDACs 4, 5, 6, 7, 9 and 10) and class IV HDAC (HDAC 11) require Zn^2+^ as a cofactor in their active site. Class III HDACs consist of sirtuins, which use NAD+ as a cofactor for their enzyme activity. HDACs play a pivotal role in cell survival, proliferation and various other key processes. The class I and IV HDACs are usually located in the nucleus. In the class II HDACs, HDACs 4, 5, 6, 7 and 9 shuttle between the cytoplasm and the nucleus and HDAC10 is predominantly located in the cytoplasm. In regard to sirtuin family, SIRT1 and 2 shuttle between the nucleus and cytoplasm. SIRT3, 4 and 5 are primarily localized to the mitochondria and SIRT6 and 7 are found in the nucleoli [4]. HDACs modify and regulate proteins involved in a broad range of cell signaling and physiological processes [1]. Further, HDACs play a pivotal role in embryonic development, proliferation and differentiation of skeletal and neural cells [5,6,7,8,9]. Hence, dysregulated activity of HDACs is implicated in many diseases [1,10]. HDAC inhibitors are small molecules designed to block the dysregulated activity of HDACs by binding to the active enzymatic sites in class I, II and IV HDACs [11,12]. Even though HDAC inhibitors have remarkable anti-tumor activity in hematological cancers and showed some responses in various diseases, they are not effective in many diseases and many patients relapse after treatment [13]. Since healthy cells are not heavily dependent on aberrantly increased HDAC activity like malignant cells are, they are significantly resistant to HDAC inhibitors-induced cell death, whereas differentiated and cancer cells are sensitive to HDAC inhibitors [3,14]. Hence, understanding exact mechanisms and effector functions of HDACs are urgently needed to selectively inhibit them and to establish a better platform to combat various malignancies in which HDACs are involved.

### 1.2. Cytokines and Chemokines

Cytokines are proteins less than 80 kDa in size, which act as messengers for cell communications. Many cell types, including T-lymphocytes, B-lymphocytes, macrophages, fibroblasts and various stromal cells produce them. Predominantly, cytokines are classified into five families, Interferons (includes Types I and II), Interleukins (IL), Tumor necrosis factor family (TNF), growth factors (Vascular endothelial growth factor (VEGF), Granulocyte macrophage colony stimulating factor (GM-CSF), etc.) and chemokines [15]. Arguably, cytokines are involved in every biological process—of which, embryonic development, stem cell differentiation, immunity, aging and disease pathogenesis are the prominent ones [16]. Since they regulate various key cellular processes, dysregulation of cytokines is involved in many human diseases [17]. Cytokines can also be regulated by reversible acetylation and deacetylation of histones, apart from other post translational modifications [18].

Chemokines (8–10 KDa) are a class of cytokines, which induce directed, concentration dependent chemotaxis in leukocytes. Major portion of the chemokine pool is produced by leukocytes and the rest by fibroblasts, endothelial and epithelial cells [19]. Currently, there are more than 45 chemokines in humans, categorized into four families based on the arrangement of cysteine residues closest to the N terminal. (1) CXC—two cysteine residues are separated by an amino acid. (2) CC—two N-terminal cysteine residues are adjacent to each other. (3) CX3C—two cysteine residues are separated by three amino acids. (4) XC—has only one cysteine residue at N-terminal end [20,21]. Chemokines bind to G protein coupled receptors and signal the transcription of a wide range of genes which encode proteins involved in inflammation, survival, apoptosis, angiogenesis, cell migration and remodeling of extracellular matrix [22]. Inflammation acts as one of the causal agents for malignant neoplastic formation and progression by promoting neoplastic cell survival, proliferation, angiogenesis and metastasis through increased release of cytokines [23,24,25]. Furthermore, dysregulated chemokine network is also documented in various other pathologies like autoimmune diseases, inflammatory diseases and vascular diseases [26].

**Figure 1:**
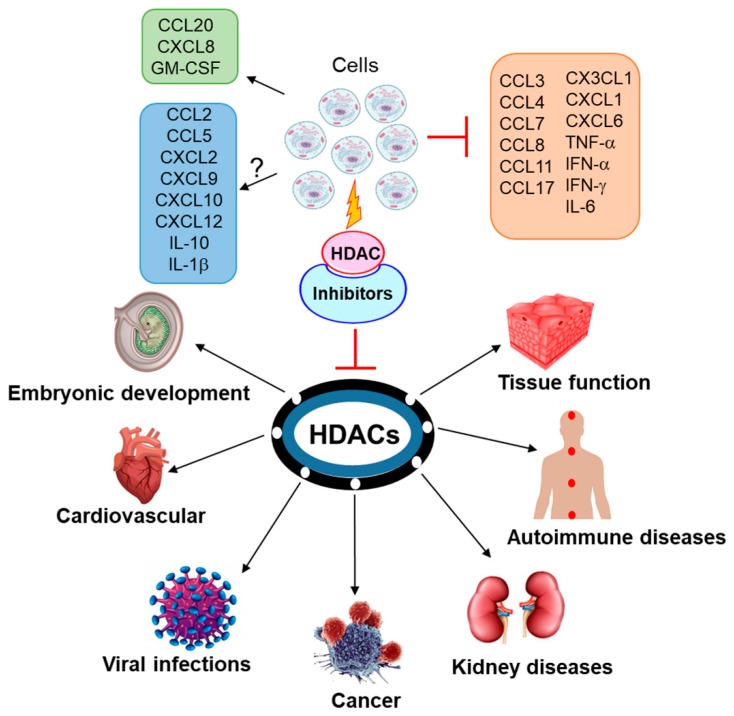
Regulation of chemokines and cytokines by histone deacetylases (HDACs) and HDAC inhibitors and implications of HDACs in various diseases. Cytokines, whose expression is inhibited on HDAC inhibition, are represented with red “T” bar. Cytokines, whose expression is induced on HDAC inhibition, are represented with black arrow. Cytokines with variable expression on HDAC inhibition are represented with an arrow and question mark.

HDAC inhibitors down-regulate several pathways involving cytokines, chemokines, growth factors and protein kinases, thereby regulating cell proliferation, apoptosis and migration [27,28]. Therefore, understanding the transcriptional regulation of cytokine expression by HDACs certainly fortifies our strategy to effectively tackle many malignancies. We have summarized cytokine and chemokine regulation by HDACs (Figure 1) and have updated the clinical status of HDAC inhibitors in various malignancies in this review (Table 1).

## 2. Cytokines, HDACs and HDAC Inhibitors

### 2.1. IFN-α

IFN-α and IFN-β are type I interferons with anti-viral, anti-tumor and immunoregulatory functions. Once activated, they induce response through Janus kinase - Signal transducer and activator of transcription (JAK-STAT) signal transduction. These cytokines regulate both innate and adaptive immune systems. HDAC1/2 are components of the Sin3A complex and HDAC activity is required for positive function for IFN-stimulated gene expression. Combined inhibition of HDAC1/2 and epigenetic reader Brd4 decreased the aberrant IFN-stimulated gene (ISG) expression in cells derived from autoimmune diseases [29]. Also, recruitment of HDAC3 to the Interferon gene promoters was shown to alter interferon gene expression [30]. Trichostatin A (TSA) treatment reportedly suppressed IFN-α induced transcriptional responses by specifically targeting IFN transcription regulation of C-terminal STAT2 transcriptional activation domain [31]. Similar results were shown with romidepsin [32].

### 2.2. IFN-γ

IFN-γ is a type II interferon mainly produced from Th1 cells and natural killer cells regulating an array of immune responses. HDAC inhibitors blocked IFN-γ-induced STAT1 phosphorylation by increasing STAT1 acetylation, reducing the transcriptional activity of STAT1 in pancreatic β-cells [33]. Similarly, HDAC inhibition was shown to repress the expression of CXCL9 and 10 through IFN-γ and STAT1 [33,34]. IFN-γ and tumor cell signaling through IFN-γ R are crucial for the anti-cancer effects of HDAC inhibitors. Alpha-galactosylceramide (α-GalCer, IFN-γ-inducing agent), increased efficacy of vorinostat against a lymphoma model in vivo [35]. Other HDAC inhibitors showed similar results, where the HDAC inhibitors decreased the expression of INF-γ in various disease models [36,37].

### 2.3. IL-1β

IL-1β modulates biological response and plays a major role in acute and chronic inflammation. HDAC inhibitors vorinostat and givinostat were shown to reduce the levels of extracellular IL-1β by preventing the exocytosis of IL-1β-containing secretory lysosomes in microglial cells [38]. However, in another study, vorinostat treatment was shown to promote LPS-induced, caspase-8 dependent IL-1β processing and secretion in human and murine dendritic cells [39]. HDAC3/6/8 inhibitor MC2625 and HDAC6-selective inhibitor MC2780 were shown to downregulate IL-1β expression in epithelial, fibroblast and myogenic cell lines and in breast silicone implant murine model [40]. The expression of cell cycle regulators p16 and p21 were changed upon treatment with romidepsin via suppression of IL-1β in an animal model of arthritis [41]. Both IL-1β and HDACs were shown to regulate matrix metalloproteases (MMPs). Wang X et al. demonstrated that treatment of human articular chondrocytes with TSA attenuated the activity of IL-1β, decreasing the expression of MMPs 1, 3 and 13. Similarly, vorinostat was shown to suppress IL-1β induced MMP-13 and TNF-α expression in osteoarthritis [42,43]. HDAC inhibition was shown to attenuate IL-1β mediated iNOS expression and NO release in pancreatic β-cells [44]. Furthermore, NF-κB activity has been shown to be regulated by HDAC inhibition, especially in relation to IL-1β signaling [33].

### 2.4. IL-6

IL-6 is a key inflammatory cytokine produced by the macrophages. HDAC inhibitor givinostat suppressed the polarization of Th17 cells and enhanced FoxP3+ regulatory T-cells, which paralleled with down-regulation of IL-6 receptor signaling in CD4+ T cells. Further, decrease of p-STAT3 and RAR-related orphan receptor T (RORγT) was reported, showing this pathway is an important target for HDAC inhibitors [45]. Similarly, treatment of congestive heart failure mice with mocetinostat improved cardiac function, decreased scar size and total collagen, which was associated with the inhibition of IL-6/STAT3 signaling pathway [46]. Selective inhibition of HDAC6 by tubastatin showed inhibition of IL-6 in paw tissues of arthritic mice model [47]. Non-specific inhibition of HDACs by vorinostat was shown to suppress the IL-6 expression through increased recruitment of CEBPα to the MCP1P1 promotor in osteoarthritis [48]. Specific knockdown of HDAC1 and 2 and use of HDAC inhibitors apicidin, MS-275, suppressed the expression IL-6 in murine microglia model [49]. Together, HDACs 1, 2 and 6 are shown to induce the expression of IL-6.

### 2.5. IL-10

IL-10 acts as an anti-inflammatory cytokine and, limits the immune response from the B cell stimulation. HDAC 11 was shown to regulate the expression of IL-10 and immune tolerance [50]. IL-10 was shown to inhibit LPS-induced CXCL8 expression in monocytes, via HDAC2 [51]. Treating murine macrophages with a pan-HDAC inhibitor LAQ824 induced chromatin changes at the IL-10 gene promoter, enabling the recruitment of HDAC11 and PU.1. This resulted in reduced IL-10 production and induction of inflammatory response [52]. Selective inhibition of HDAC6 in APCs resulted in decreased STAT3 phosphorylation, as well as reduced STAT3 recruitment to the IL-10 gene promoter region [53]. Supporting these studies, mycobacterium tuberculosis infection is reported to dysregulate HDAC6/HDAC11 levels to induce IL-10 in macrophages [54]. However, few studies have reported that HDAC inhibitors increase the expression of IL-10, suggesting that the regulation of HDAC inhibitors on IL-10 needs to be evaluated based on context [40,55].

### 2.6. TNF-α

TNF family is involved in numerous biological process including apoptosis and inflammation. TNF signaling entails activation of nuclear factor kappa-light-chain-enhancer of activated B cells (NF-κB) and Mitogen activated protein kinases (MAPKs), resulting in the activation of transcription factor AP1, which promotes cell survival of malignant cells [56]. Elevated HDAC activity was shown to increase TNF-α expression through various mechanisms [57,58,59]. TNF-α-related apoptosis-inducing ligand (TRAIL) was shown to exhibit antitumor activity in a variety of tumor cells. TRAIL in combination with HDAC inhibitors was shown to block cell cycle and increase apoptosis in various cancers [60,61,62]. In addition, Wang X et al. reported that Th1 immunity associated pro-inflammatory cytokines, TNF-α, IL-12 and IFN-γ were enhanced by HDAC6 inhibition [63]. However, another study showed HDAC6 inhibitor inhibited TNF-α and IL-6 in LPS stimulated human THP-1 macrophages [47], suggesting that TNF-α regulation by HDACs varies across cell types.

### 2.7. GM-CSF

GM-CSF recruits and activates various leucocytes and acts as a hematopoietic growth factor. Inhibition of HDAC activity in alveolar macrophages using TSA was shown to increase the release of GM-CSF [64]. Similarly, low dose HDAC inhibitors increased the release of GM-CSF from acute myeloid leukemia cells [65] and class I HDAC inhibition increased its expression in fibroblasts [66]. Specifically, HDAC1 [67] and HDAC2 [68] are shown to represses the expression of GM-CSF. In addition, HDAC2 mediated deacetylation of glucocorticoid receptor was shown to suppress the expression of GM-CSF through its binding with NF-κB [69]. Taken together, inhibition of HDAC activity, HDAC1 and 2 specifically, increases the release of GM-CSF, increasing the immune response.

## 3. Chemokines, HDACs and HDAC Inhibitors

### 3.1. CCL2

CCL2, also known as MCP-1 plays a major role in recruiting lymphocytes, monocytes and neutrophils by inducing chemotaxis. Across different kinds of cells, CCL2 expression was shown to be regulated by HDACs 1, 2, 3 and 11. HDAC1 in association with RNA pol II [70], HDAC2 - complexed with YY-1, HDAC3 along with SP-1 and c-jun [71] and HDAC11 with PU-1 [72] were shown to induce the expression of CCL2. However, few reports showed repressor role of HDACs in the context of CCL2 expression. HDAC1 in association with NF-κB p50 [67] and HDAC3 with NCOR [73] were shown to bind to the promoter, repressing its expression. Furthermore, Non-specific HDAC inhibition induced the expression of CCL2 in B6 melanoma [74]. Taken together, the expression of CCL2 mediated by HDACs is dependent on the transcriptional activator or repressor complex in which, HDACs are a part of.

### 3.2. CCL3

CCL3 is also known as macrophage inflammatory protein 1α and is known to induce potent inflammatory response by recruiting neutrophils. CUDC-907, a dual P13K and HDAC inhibitor was shown to increase the acetylation of H3 at promoters of various genes. However, it decreases extracellular-signal-regulated kinase (ERK), MAPK/ERK kinase (MEK) and STAT3 phosphorylation, decreasing the expression of T cell chemokines CCL3, 4, 17 and 22. This suggests that HDAC inhibition at promoter alone is not sufficient to increase gene expression but modulation of signaling mediators and transcription factors play a crucial role [75].

### 3.3. CCL4

CCL4, also known as macrophage inflammatory protein 1β, was shown to induce the release of other pro-inflammatory cytokines such as IL-1, IL-6 and TNF from fibroblasts and macrophages. Choi et al. showed that CKD 506, a specific HDAC6 inhibitor decreased CCL4 kidney levels [76]. In addition, elevated levels of CCL4 and inflammation in Alzheimer’s brain was decreased by HDAC inhibition using sodium butyrate, suggesting that HDAC inhibitors can be used to ameliorate neurodegenerative phenotypes [77]. Together, CCL4’s expression decreases with HDAC inhibition.

### 3.4. CCL5

CCL5 (RANTES) is primarily expressed and released because of NF-κB activity and is responsible for homing T lymphocytes, eosinophils, basophils and macrophages. As shown by Topper et al. in lung tumors, CCL5 expression is controlled by methylation as well as acetylation at its promoter. Inhibition of both by 5-azacytidine and givinostat increased its expression [78]. PD-1 blockade along with HDAC inhibition increased plasma levels of CCL5 in B16 melanoma tumors too [74]. However, specific HDAC1 inhibition in intestinal epithelial IEC-6 cells decreased CCL5 expression. But, HDAC1 binding partners at CCL5 promoters remains to be explored [70].

### 3.5. CCL7

CCL7 is also known as monocyte chemotactic protein 3 (MCP-3) and is a strong chemotactic protein for various kinds of leukocyte. Expression of CCL7 is predominantly mediated by PU-1. HDAC inhibition decreases the nuclear presence of PU-1, thereby decreasing CCL7 expression in bone marrow macrophages [79]. However, regulation of CCL7 expression by individual HDACs remains to be explored.

### 3.6. CCL8

CCL8 is also known as monocyte chemotactic protein 2 (MCP-2) and acts through CCRs 1, 2, 3 and 5 to induce chemotaxis in leucocytes. Entinostat, a HDAC1 and 2 inhibitor decreased the secretion of CCL8 from E11 rheumatoid arthritic synovial fibroblastic cells, suggesting that either or both of the HDACs increase the expression of CCL8 in E11 cells [80]. However, the transcription factors responsible for entinostat mediated decrease in CCL8 expression are unknown.

### 3.7. CCL11

CCL11 is also known as eosinophil chemotactic protein for its role in selectively recruiting eosinophils, as a part of allergic response. TSA, a nonspecific HDAC inhibitor, decreased the infiltration of lungs with eosinophils, lymphocytes and neutrophils by decreasing the expression of eosinophil recruiting chemokines CCL 11 and CCL 24. Along with the above chemokines, TSA also decreased the expression of IL-5 and IL-13, decreasing type 2 innate immune response mediated by group 2 innate lymphoid cells [81], suggesting that HDACs promote the expression of these cytokines.

### 3.8. CCL17

CCL17 is also known as thymus and activation regulated chemokine (TARC). It is one of the TH2 lineage chemokines and its expression and secretion was decreased by non-specific HDAC inhibition with vorinostat in Hodgkin lymphoma cells. Vorinostat skewed the cytokine release profile more towards TH1 type response by inhibiting the phosphorylation and mRNA levels of STAT6, suggesting that the anti-tumor activity of HDAC inhibitors is also mediated by immunoregulatory mechanisms [82]. However, we currently do not have the data regarding CCL17 expression by individual HDACs.

### 3.9. CCL20

CCL20 is also called as macrophage inflammatory protein-3 and it attracts leucocytes by acting through CCR6. In CaCo-2 intestinal epithelial cells, HDAC inhibition increased Shanchol^TM^ (whole cell cholera vaccine) mediated CCL20 expression. Additionally, co-treatment with HDAC inhibitor butyrate increased the chemotactic migration of immature dendritic cells, increasing mucosal immune response [83]. The above results suggest that HDACs repress the expression of CCL20, decreasing the immune response.

### 3.10. CX3CL1

CX3CL1 consists of 373 amino acids and is the only chemokine present in CX3C chemokine family. Novitskaya et al. in ischemia reperfusion kidney model, showed a decrease in macrophage infiltration mediated by decreased CX3CL1 levels on HDAC inhibitor treatment [84]. In addition, Zhou et al. showed that CX3CL1 expression is controlled by HDACs and NF-κB in epithelial cells. However, HDACs regulate CX3CL1 levels through a secondary mechanism where HDACs inhibit the expression of miR 424 and 503, which in turn inhibit CX3CL1 expression [85]. Taken together, HDACs positively regulate CX3CL1 expression both through primary and secondary mechanisms.

### 3.11. CXCL1

CXCL1 promotes inflammation by recruiting and activating neutrophils to the site. Silencing HDAC1 decreases basal levels of CXCL1 in intestinal epithelium [70]. In addition, HDAC interacts with adenovirus small E1a protein, p400, lysine acetyl transferase p300 and acetylated RB1, which is locked into repressor confirmation to increase the stability of mRNAs of CXCL1 and 2 in HeLa cells transfected with adenovirus [86]. The above reports suggest that HDACs, especially HDAC1 promotes the expression of CXCL1.

### 3.12. CXCL2

CXCL2 mobilizes leucocytes by acting through CXCR2. Its expression was shown to be regulated by HDACs 1, 2 and 3 in various kinds of cells. HDAC3 is shown to remove the inhibitory acetylations on NF-κB at K122, 123, 314 and 315, promoting the expression of CXCL2 [87]. In pancreatic cells, HDAC2 silencing increased the expression of CXCL2 and HDAC1 silencing decreased the levels, suggesting that HDAC2 represses the expression, whereas HDAC1 promotes it [88]. However, in intestinal cells, HDAC1 silencing increases IL-1β mediated CXCL2 expression through phosphorylation of p65 at S536 [70], suggesting that HDACs tend to regulate the expression of CXCL2 based on the signal, binding to transcription factors, their post translational modifications and the cell type.

### 3.13. CXCL6

CXCL6 is also known as Granulocyte chemotactic protein-2. Class I or non-specific HDAC inhibition with entinostat and vorinostat respectively decreased CXCL6—granulocyte chemotactic protein-2’s expression. In addition, HDAC inhibitor decreased the LPS induced GCP-2, MCP-2 and MIF expression, suggesting that HDACs promote CXCL6 gene expression either as a part of the transcription factor complex or through their effects on the transcription factors itself. However, the coordination in between specific HDACs and transcription factors at the CXCL6 promoter remains to be explored [80].

### 3.14. CXCL8

CXCL8 is a proangiogenic chemokine known to promote tumor cell proliferation, survival and migration [89,90,91] and is known to act through CXCRs 1 and 2 [92]. Induction of CXCL-8 expression and release by HDAC inhibition in ovarian cancer decreased their efficacy. HDAC inhibitor induced IL-8 expression is dependent on IKK mediated NF-κB p65 recruitment and its acetylation at lysine 314 [93,94]. Chavey et al. in estrogen receptor-positive MCF-7 breast cancer cells showed that HDAC inhibition with TSA increased IKK and NF-κB dependent CXCL-8 expression [95]. In addition, class I HDACs but not class II HDACs regulate CXCL-8 expression in ovarian cancer [96]. In agreement, Castellucci et al. have shown that IL-10 decreases LPS mediated CXCL-8 expression by decreasing S276P-p65 and increasing HDAC2’s binding to CXCL-8 promoter in monocytes [51]. Bartling et al. documented that reduction in cystic fibrosis transmembrane conductance regulator (CFTR) in cystic fibrotic airway results in decreased levels of HDAC2 in bronchial epithelium, which is in parallel to increased CXCL-8 expression in the same [97]. Taken together, CXCL8 expression is predominantly mediated by class I HDACs.

### 3.15. CXCL9

CXCL9 is also known as Monokine Induced by Gamma Interferon (MIG). In rheumatoid arthritis fibroblast like synoviocytes, Angiolilli et al. showed that HDAC3 inhibition induces the expression of CXCL9 and 11 [98]. In addition, they went on to show that downregulation of HDAC5 also results in the induction of CXCL9, 10 and 11 [99]. However, HDAC inhibition using givinostat or HDAC 1,2 and 3 inhibition in pancreatic INS-1 cells decreased interferon (IFN)-γ and IL-1β induced CXCL9 expression [33]. Further, Inhibition of HDACs by TSA or vorinostat in myeloid dendritic cells decreased the TH1 attracting cytokines CXCL9, 10 and 11 skewing the balance towards TH17 recruiting cytokines [100]. These reports suggest that CXCL9 expression is signal and cell type dependent and is regulated by HDACs 1, 2, 3 and 5.

### 3.16. CXCL10

CXCL10 is also known as Interferon gamma-induced protein 10 (IP-10). HDACs 1 [67] and 4 [101] were shown to repress the expression of CXCL10 in hepatic cells. When stimulated by IL-1β [102] and IFN-α [103], HDAC inhibition was shown to increase CXCL10 expression. However, when stimulated by IFN-γ in HeLa cells [104], IL-1β in pancreatic cells [33] and TNF-α in endothelial cells [105], HDAC inhibition was shown to decrease CXCL10 expression, suggesting that the signal pathways and transcription factor—HDAC complex dictates the roles of HDACs in CXCL10 expression.

### 3.17. CXCL12

CXCL12 (stromal cell-derived factor 1 - SDF1) is a potent chemokine, which acts through CXCR4. Class I HDAC inhibition using apicidin decreased its expression in pulmonary fibroblasts, demonstrating that class I HDACs induces CXCL12 expression [66]. However, in colon cancer cells, Romain et al. observed that non-specific HDAC inhibition by vorinostat, valproic acid, butyrate increased CXCL12 expression, which was otherwise decreased in both MSI and MSS colon cancer tumors, warranting more studies on the roles of specific HDACs on CXCL12 expression [106].

## 4. HDAC Inhibitors in Cancer

Aberrant expression of HDACs is found in various cancers. High expression of HDACs is associated with poor prognosis and survival [107]. Several structurally unique HDAC inhibitors including hydroxamates, benzamides, cyclic peptides and aliphatic acids were developed to block the malignant function of HDACs [108]. Apart from targeting histones, HDAC inhibitors reportedly target several molecules. About 5–10% of genes can be altered by HDAC inhibitors and myriad different mechanisms have been reported in regards to their mode of action [11].

To date, four HDAC inhibitors have been approved as second line treatment for relapsed peripheral T-cell lymphoma (PTCL) and/or cutaneous T-cell lymphoma (CTCL) and multiple myeloma by the FDA [109]. The first approved HDAC inhibitor (2006) was vorinostat (SAHA, Zolinza) for CTCL patients. Vorinostat is a hydroxamic acid derivative and is structurally related to TSA [110]. Favorable preclinical studies and phase II and IIa results in CTCL patients led to a phase IIb open-label, multi-center trial in patients with IB-IVA Sézary syndrome. This study enrolled seventy four patients who have received a minimum two prior systemic therapies. The objective response rate was 29.7% overall and 29.5% in Sézary syndrome patients (stage IIB or higher), with one patient achieving complete response. Pruritus relief was found in thirty five patients, including responders and non-responders [111].

Romidepsin is a cyclic tetrapeptide (Istodax, FK228, FR901228, depsipeptide) approved for treatment of CTCL in 2009 and for PTCL in 2011. Romidepsin was isolated from a gram-negative bacterium *Chromobacterium violaceum*. [110]. A response rate of 34% was reported in a phase II study conducted in 96 patients with stage IB to IVA CTCL, where five patients showed complete response [112]. In the relapsed or refractory PTCL phase II trial consisting of 131 patients, a response rate of 25% (33 of 130) was reported, including nineteen complete responses [113].

In 2014, belinostat (Beleodaq, PXD-101), a hydroxamic acid-type HDAC inhibitor was approved for the treatment of relapsed or refractory PTCL patients [110]. In a phase II study conducted in 120 toxicity evaluable PTCL patients, the response rate was 26% and 13 complete responses were reported [114].

In 2015, panobinostat (LBH-589, Farydak), a cinnamic hydroxamic acid was approved by FDA for the treatment of relapsed and refractory multiple myeloma in combination with bortezomib and dexamethasone. In a phase III trial conducted in 768 adult multiple myeloma patients, median progression free survival was significantly increased in the panobinostat combination treatment [115].

Apart from these, there are also other HDAC inhibitors which target different class of HDACs, which are currently being evaluated in many phase II/III clinical trials (Table 1). Further, preclinical studies showed HDAC inhibitors synergies with a variety of anti-cancer agents against a wide range of cancers with different mechanisms of action [14,116,117].

## 5. HDAC Inhibitors in Viral Infections

HDACs play paradoxical roles during virus infection. During acute phase infection, HDACs restrict the virus, whereas in HIV and herpes virus infection they promote latency. HDAC inhibitors have been exploited in reactivating latent viral infections [118,119,120].

### 5.1. HIV

Antiretroviral therapy (ART) cannot eliminate the latent HIV in long-lived cells, which is a major roadblock to cure HIV [121]. HDACs were shown to be associated with transcriptional regulation of HIV promoter region and promote HIV latency. Both in vitro and in vivo results showed that HDAC inhibitors can reactivate latent HIV by inhibiting HDACs [122]. In phase I/II clinical trial, vorinostat was tested in 11 HIV-infected patients, treated previously with ART. Vorinostat treatment showed a significant-fold increase of HIV mRNA expression in CD4+ T cells [123]. Similarly, in another study 16 HIV-infected aviremic individuals were treated with interval dosing of vorinostat. This dosing schedule was well tolerated by patients and resulted in an increase in HIV RNA expression CD4+ T cells [124]. In a phase I/II HIV clinical trial, panobinostat was administrated in 15 patients. Panobinostat treatment disrupted HIV latency and other study showed that panobinostat treatment was not associated with adverse CNS effects on HIV patients on suppressive ART [125,126]. These results show HDAC inhibitors show some clinical activity and they need to be combined with novel combinations to improve treatment efficiency for HIV. In Phase I/II clinical trial NCT02471430, HIV-1 infected patients treated with suppressive combination antiretroviral combination therapy (cART) are currently being investigated in combination treatment with panobinostat and the immunomodulatory cytokine IFN-α2α.

### 5.2. DNA Virus

Treatment with TSA enhanced hyperacetylation of the viral promoter region in the fatal viral disease leukoencephalopathy, caused by human polyoma JC virus [127]. Likewise, sirtuins exhibit antiviral properties against varicella zoster virus, herpes simplex virus and vesicular stomatitis virus [128,129,130]. Yu et al. reported elevated sirt2 promotes Hepatitis B virus (HBV) replication and, sirt2 inhibitor AGK2, resulted in a decline in HBV replication both in vitro and in vivo due to reduced promoter activity [131].

During herpes simplex virus 1 (HSV-1), human cytomegalovirus (HCMV), Kaposi’s sarcoma associated herpesvirus (KSHV) and Epstein-Barr virus (EBV) infections, the acetylation/deacetylation status of the viral promoter were shown to be the determining factor for promoting viral latency and this effect was mediated by HDACs. HDAC inhibitor reactivated the latent virus in Kaposi’s sarcoma induced herpes virus infection [132,133,134].

HDAC inhibitors have been employed as attractive targets for enhancing anti-tumor activity in oncolytic viruses. In the study conducted by Nakashima et al. the immune response against glioma cells was accelerated by delivering oncolytic HSV-I. Most interestingly, the oncolytic activity of the virus was enhanced by inhibiting the activity of HDAC6, indicating an important role of this HDAC in breaking latency [135].

The deacetylase activity of SIRT1 was inhibited using a small molecule inhibitor EX-527, which resulted in decrease in HCMV. This study demonstrated the anti-viral role of sirt1 and inhibiting its activity proved detrimental to the host [136].

### 5.3. RNA Virus

Husain et al. demonstrated the antiviral role of HDAC6, inhibiting influenza A virus (IAV) infection in A549 cells. Notably, inhibition of HDAC6 activity by tubacin increased the viral titer, highlighting the role of host HDAC6 in regulating IAV infection [137]. In a similar study, Nagesh et al. demonstrated the antiviral role of class I HDACs, HDAC1 and HDAC2 against IAV infection [138,139]. IAV infection decreased the acetylated histone H3 levels indicating impaired HDAC activity. Further, when they inhibited HDAC activity by TSA, there was a significant increase in the viral titer suggesting an anti-IAV role for class I HDACs [138]. These studies indicate that inhibition of HDAC activity by TSA might result in deleterious effects to the cells as they promote the virus infection, at least in the context of IAV. Upon EX-527 mediated inhibition of SIRT1 activity in HCMV infection, there was a decrease in the IAV infection, suggesting a protection of SIRT1 across different types of virus [136].

Contrastingly, in the fatal dengue virus infection, macrophages treated with HDAC inhibitor VPA decreased the cytokine production in the infected cells, indicating a novel therapeutic aspect of HDAC inhibitor in treating dengue virus infections [140].

During west Nile virus infection, the combination of NITD008 (inhibitor of RNA polymerase in Flavivirus) with vorinostat during CNS phase I infection significantly improved the disease outcome by reducing inflammation and neuronal death [141].

## 6. HDAC Inhibitors in Cardiovascular Diseases

Cardiovascular diseases are the leading cause of death in the world despite many therapeutic advances [142]. Although HDAC inhibitors have shown positive effects in preclinical models of cardiovascular diseases, cardiac side effects such as, QTc prolongation, hypotension, myocardial infarction and so forth were reported in cancer patients upon HDAC inhibitor treatment [143], therefore HDAC inhibitors were not evaluated in cardiovascular patients. Here we have summarized the malignant role of HDACs and the potential of HDAC inhibitors reported in preclinical models of cardiovascular disorders.

### 6.1. Cardiac Hypertrophy

Cardiac hypertrophy is an adaptation of the cardiac myocytes resulting from the increased hemodynamic load due to underlying conditions such as hypertension, vascular disease and myocardial infarction [144]. Cao et al. demonstrated that the pathological cardiac hypertrophy, characterized by cardiomyocyte autophagy was HDAC1 and HDAC2 dependent. Inhibition of class I HDAC activity by TSA and a panel of structurally different HDAC inhibitors attenuated pathological cardiac hypertrophy [145]. Another report has also highlighted the role of class I HDACs in mediating cardiac hypertrophy by activation of mTORC11 pathway and the growth of cardiomyocytes. Strikingly, class I HDACs inhibition by apicidin, resulted in an increase of TSC2, an inhibitor of mTORC1. Decrease in mTORC1 resulted in reduced cardiomyocyte growth and decreased hypertrophy [146]. During hypertrophy, pressure overload induced cardiac hypertrophy was associated with a marked increase in the histone acetylation of genes involved in extracellular matrix deposition, inflammation and cardiac contraction. Most importantly, NF-κB driven cytokine expression was enhanced during hypertrophy and this effect was reversed with HDAC inhibitor TSA [147]. In DOCA-salt hypersensitive rats, sodium valproate mediated inhibition of HDAC activity resulted in specific reduction of HDAC6 and HDAC8 activity. This study suggested a specific role of HDAC6 and HDAC8 under hypertension induced cardiac hypertrophy [148].

### 6.2. Cardiac Fibrosis

After an acute cardiovascular insult, the cardiomyocytes undergo a process of cardiac remodeling characterized by fibrosis, which is beneficial [149,150]. However, if unchecked, this process can cause excessive inflammation, oxidative stress, resulting in cardiac ischemia, which becomes detrimental. In response to a cardiac insult, HDAC activity is reported to be upregulated, resulting in epigenetic modifications and altered gene expression of cardiac cells. Milan M et al. reported that inhibition of HDAC activity by givinostat prevented pathological cardiac fibrosis by reducing the inflammatory cytokines which is reflected on decreased epithelial-mesenchymal transition [151]. In a separate study, it was shown that diabetic mice exhibit poor cardiac function and most interestingly, this effect was reversed upon treatment with the HDAC inhibitor. Treatment with sodium butyrate reduced the levels of cardiac hypertrophy and increased angiogenesis [152]. The European society of cardiology and the American heart association revisited the severity of heart failure with preserved ejection fractions (HFpEF). The clinical outcomes of HFpEF from the past two years with heart failure with reduced ejection fraction (HFrEF) were summarized. It was surprising to note that conventional therapeutic intervention using beta-blockers, angiotensin converting enzyme inhibitors/angiotensin receptor blockers or aldosterone-antagonists did not adequately reduce the burden of HFpEF. In this context, a rodent HFpEF study suggested that HDAC inhibition prevent HFpEF and restores normal heart conditions. This report gave a very promising application of HDAC inhibitor in treating HFpEF, at least pre-clinically [153].

### 6.3. Myocardial Infarction

A decrease or loss of blood supply to certain regions of the heart results in congestive heart failure or myocardial infarction, which is characterized by ischemia and damaged heart muscles. Congestive heart failure characterized by interstitial fibrosis, cardiac remodeling and acute myocardial infarction are mediated by HDAC1 and HDAC2. Importantly, inhibition of class I HDAC activity by mocetinostat improved cardiac function by improving the ventricular contractility and reduced fibrosis in animal models for heart failure. Previous studies have demonstrated the role of TSA in retarding cardiac fibrosis immediately after cardiac injury. However, the above described study used specific class I HDAC inhibitor, mocetinostat after development of fibrosis and this suggests a potential therapeutic application of mocetinostat [154]. Despite the contradicting role of HDAC inhibitor in acute coronary artery disease, administration of tributyrin, VPA and TSA have reduced the scar size of MI, prevented cardiac dysfunction and repressed cardiac remodeling [155,156,157].

### 6.4. Atherosclerosis

Atherosclerosis is a macro vascular complication characterized by accumulation of lipid-laden macrophages on the arterial walls, resulting in further recruitment of inflammatory macrophages, contributing to the progression of the disease [158]. Several inflammatory cytokines secreted by the atherosclerotic plaque macrophages accelerate the development of the disease. Scriptaid or TSA mediated HDAC inhibition resulted in reduced neointima formation during the disease progression. In contrast, other reports indicate that HDAC inhibitor stimulate the progression of atherosclerosis. Of note, HDAC inhibitor effectively suppresses vascular smooth muscle cell proliferation, which could substantially reduce the progression of the disease. It is important to note that the concentration of HDAC inhibitor used might have differential effect on the disease. For instance, a low dose TSA account for anti-inflammatory properties whereas a high dose TSA contributes to pro-inflammatory phenotype. Hence, a relatively ideal strategy would be to target a specific HDAC to have a specific effect. However, this still remains a great challenge [109].

### 6.5. Cardiac Arrhythmia

Cardiac arrhythmia is characterized by an irregular rhythm of heartbeat, which could be either too slow or too fast. Several factors contribute to arrhythmia including but not limited to coronary artery disease, diabetes stress, smoking, hypertension, cardiac hypertrophy and genetics [159]. Very few studies have tried to identify the role of HDACs in treating arrhythmia as few of the risk factors have known to be reduced by HDAC inhibitor treatment. A study revealed that treatment with TSA dramatically corrected arrhythmia and they hypothesized that this effect is mainly mediated by recruitment of HDAC2 by homeodomain protein homeobox (HopX) and this axis mediates altered arrhythmia [109]. Another study also showed that myocyte specific over expression of HDAC2 dysregulated the key genes involved in ion channel maintenance and this could contribute to abnormal heart rhythm leading to arrhythmia [160,161].

## 7. HDAC inhibitors in Autoimmune Diseases

HDACs were reported to be upregulated in autoimmune diseases leading to increased nuclear translocation and binding of the transcription factors. Predominantly, HDACs were shown to effect STAT3 and NF-κB pathways, leading to activation of pro-inflammatory genes. HDAC inhibitors have been proven to be effective in several pre-clinical models of autoimmune diseases such as rheumatoid arthritis, systemic lupus erythematosus, multiple sclerosis, systemic sclerosis, psoriasis and ulcerative colitis [162].

### 7.1. Rheumatoid Arthritis

Rheumatoid arthritis is characterized by progressive destruction of the affected joints. HDAC inhibitors (phenylbutyrate, TSA and romidepsin) reduced the effect of inflammation and bone destruction in animal models of rheumatoid arthritis [41,163]. Rheumatoid arthritis fibroblast-like synoviocytes (RA FLSs) obtained from arthritis patients when treated with vorinostat induced apoptosis via generation of ROS and suppressed NF-κB activation and anti-apoptotic proteins (Bcl-xL and Mcl-1) [164]. TSA inhibited cell viability and reduced the expression of MMP-2, MMP-9, PI3K and p-Akt in RA FLSs [165]. HDAC6 inhibitor, CKD-L decreased the arthritis score in collagen-induced arthritis model and reduced the expression of TNF-α and IL-1β in rheumatoid arthritis patients. Mice treated with CKD-L increased CTLA-4 expression and the suppressive function of Treg cells [166]. In addition, cytokine production was shown to be suppressed by HDAC inhibitor in RA FLSs [167,168].

### 7.2. Multiple Sclerosis

Multiple sclerosis is a chronic disorder of central nervous disease involving gliosis, demyelination and neuronal loss [169]. Transcriptional imbalance contributes to pathology of multiple sclerosis. TSA treatment reduced the expression of various genes in experimental autoimmune encephalomyelitis, a model of multiple sclerosis [170]. Dimethyl fumarate (methyl ester of fumaric acid), approved to treat adults with relapsing multiple sclerosis was shown to modify expression of HDACs in primary rat astrocytes, which potentially could contribute to Nrf2 activation, suppressed inflammatory responses and caused changes in gene expression [171]. Vorinostat was shown to suppress dendritic cell and dendritic cell-mediated Th1 and Th17 cell functions in experimental autoimmune encephalomyelitis in vivo [172].

### 7.3. Ulcerative Colitis

Ulcerative colitis and Crohn’s diseases are inflammatory bowel disorders of the intestinal tract caused by dysfunction of the immune system, genetic and epigenetic alterations. Valproic acid, vorinostat and givinostat, were shown to reduce pathology in murine colitis models, through reduction of pro-inflammatory cytokine release, regulating cell death, transcription factors and modulation of HDAC-regulatory miRNAs [173]. Vorinostat was shown to decrease inflammatory changes in dextran sulfate sodium (DSS)-induced colitis by suppressing local secretion of pro-inflammatory cytokines, chemokines and mobilization of inflammatory cells [174]. Similarly, givinostat was shown to attenuate inflammation-mediated tumor growth in DSS-induced colitis mice [175]. HDAC6 inhibitor treatment significantly decreased DSS-induced colitis in mice and showed a better protective effect than mesalazine, a commonly used drug in the clinic [176]. Treg cells have been reported to be crucial for development of colitis. HDAC9 inhibition increased Foxp3 gene expression, as well as the production and suppressive function of Treg cells in in vivo [177].

### 7.4. Systemic Lupus Erythematous (SLE)

SLE is characterized by the production of antibodies against its own cells. HDAC inhibitors have been shown to regulate expression of multiple genes involved in SLE [178]. Aberrant HDAC levels were reported in animal model of lupus and SLE patients [179,180]. Selective inhibition of HDAC6 by ACY-738 decreased disease pathogenesis in lupus animal model [181]. Similar results were shown by Regna NL et al. [179]. HDAC inhibitors TSA, vorinostat, panobinostat and givinostat were shown to be effective in lupus models in-vivo [180].

## 8. HDAC Inhibitors in Kidney Diseases

HDAC inhibitors have shown to be effective in reducing the pathogenesis in pre-clinical models of several kidney diseases including, glomerulosclerosis, tubulointerstitial fibrosis, lupus nephritis, polycystic kidney disease, renal injury and diabetic Nephropathy [4,182].

HDAC inhibitors as single agents and in combinations are currently being evaluated in renal cell carcinoma (RCC) clinical trials [4]. Preclinical studies have shown that entinostat in combination with IL-2 exhibited synergistic antitumor effect in RCC bearing mice. Further, the combination down- regulated Foxp3 expression and function of Tregs [183]. Phase I/II clinical trial of entinostat in combination with IL-2 on 47 metastatic RCC patients showed promising clinical activity. An objective response rate of 37%, median progression-free survival of 13.8 months and the median progression-free survival was 65.3 months was reported. Further, decreased numbers of Tregs after entinostat treatment were associated with response (*p* < 0.05) [184]. High dose IL-2 in combination with entinostat is currently being investigated in advanced RCC patients in phase II clinical trials (NCT03501381 and NCT01038778). Entinostat enhanced the anti-tumor effect of immune check point PD-1 inhibitor in RCC xenografts by inhibition of myeloid-derived suppressor cells. Further, significant alterations in cytokine/chemokine release was observed with a transition away from an immune-suppressive tumor microenvironment [185]. Entinostat is currently being evaluated in Phase I/II clinical trial (NCT03024437) in combination with atezolizumab (anti PD-L1 antibody) and bevacizumab (anti-VEGF) in advanced RCC patients. In another phase I clinical trials (NCT02909452 and NCT02619253) entinostat/vorinostat in combination with pembrolizumab (anti-PD-1) are being evaluated renal neoplasms. Entinostat is also being evaluated in combination with both PD-1 inhibitor (nivolumab) and CTLA-4 inhibitor (ipilimumab) in phase II clinical trial (NCT03552380) in previously untreated RCC. HDAC inhibitor abexinostat in combination with FDA approved VEGF inhibitor pazopanib showed promising and prolonged durable responses in metastatic RCC patients [186,187] and is been currently evaluated in phase III clinical trial (NCT03592472).

## 9. Conclusions

In this review, we have highlighted the growing importance of the regulation of cytokines and chemokines by HDACs and HDAC inhibitors in various human diseases. Though HDAC inhibitors are approved by FDA in hematological cancers and have shown signs of clinical activity in inflammatory disorders and viral infections, many patients relapse after treatment. Most HDAC inhibitors target wide range of proteins, which makes it difficult to identify specific targets and to assess whether their biological and clinical effects are due to the inhibition of an individual HDAC or the combined inhibition of multiple HDACs and protein complexes. Since cytokines play a key role in immunity and dysregulation and are implicated in many human disorders, understanding the role of HDACs and HDAC inhibitors in the perspective of how they regulate cytokine and chemokine expression can lead to novel combinations to treat human diseases.

## Figures and Tables

**Table 1:** HDAC inhibitors clinical status. The clinical trials information was obtained from https://clinicaltrials.gov/, accessed on: 31 January 2019.

HDAC Inhibitor	Synonym	Specificity	Clinical Status
Vorinostat	SAHA	pan-HDAC	FDA approved for cutaneous T-cell lymphoma (CTCL)
Belinostat	PXD101		FDA approved for peripheral T-cell lymphoma (PTCL)
Panobinostat	LBH589		FDA approved for multiple myeloma
Trichostatin A	TSA		Not tested
Givinostat	ITF2357		Phase II—in combination With Hydroxyurea in Polycythemia Vera (NCT00928707), phase II—chronic myeloproliferative neoplasms (NCT01761968), phase II/III—duchenne’s muscular dystrophy (NCT03373968) and phase II—juvenile idiopathic arthritis (NCT01261624)
Resminostat	RAS2410, 4SC-201		Phase II—in combination with sorafenib in advanced hepatocellular carcinoma (NCT00943449 and NCT02400788), phase I/II—advanced colorectal carcinoma (NCT01277406) and phase II—refractory Hodgkin’s lymphoma (NCT01037478) and phase II—sézary syndrome (NCT02953301)
Quisinostat	JNJ-26481585		Phase II—combination with chemotherapy in ovarian cancer (NCT02948075) and phase II—cutaneous T-cell lymphoma (NCT01486277)
Abexinostat	PCI-24781		Phase I/II—in combination With doxorubicin to treat sarcoma (NCT01027910), phase II—relapsed/refractory follicular lymphoma (NCT03600441) and phase III—combination with pazopanib in metastatic renal cell carcinoma (NCT03592472)
Romidepsin	FK228, desipeptide	Class I	FDA approved for PTCL and CTCL
CHR-3996			Phase I/II—EBV-associated lymphoid malignancies (NCT03397706)
Entinostat	MS-275		Phase II—in combination with azacitidine in breast cancer, colorectal cancer, chronic myelomonocytic leukemia or acute myeloid leukemia and non-small cell lung cancer (NCT01349959, NCT01105377, NCT00313586, NCT00387465), phase II—metastatic melanoma (NCT00185302), phase II—refractory Hodgkin’s lymphoma (NCT00866333), phase II—non-small cell lung cancer (NCT00750698) and phase III—in combination with exemestane in ER/PR+ breast cancer (NCT02115282)
Tacedinaline	CI994		Phase III—in combination with gemcitabine in non-small cell lung cancer (NCT00005093), phase II—in combination with gemcitabine in pancreatic cancer (NCT00004861) and phase II—advanced myeloma (NCT00005624)
Domatinostat	4SC202		Phase I/II—advanced melanoma, non-responders to anti-PD-1 therapy (NCT03278665)
Ricolinostat	ACY-1215	Class II	Phase I/II—with various combination in multiple myeloma (NCT01997840, NCT01583283 and NCT01323751)
Valproic acid	VPA	Classes I and IIa	FDA approved for seizures and manic-depressive disorders
Mocetinostat	MGCD0103	Classes I and IV	Phase II—urothelial carcinoma (NCT02236195), phase II—combination with gemcitabine in metastatic leiomyosarcoma (NCT02303262), phase II—relapsed and refractory lymphoma (NCT00359086), phase II—refractory chronic lymphocytic leukemia (NCT00431873) and phase II—combination with myelodysplastic syndrome or acute myelogenous leukemia (NCT00324220)
Pracinostat	SB939	Classes I, II and IV	Phase II—in combination with ruxolitinib in myelofibrosis (NCT02267278), phase II—myelodysplastic syndrome (NCT01993641), phase II—combination with azacitidine in myelodysplastic syndrome (NCT01873703), phase II—combination with azacitidine in acute myeloid leukemia (NCT01912274), phase II—in patients myelofibrosis (NCT01200498) and phase II—metastatic sarcomas (NCT01112384)
Sirtuin inhibitors		SIRT family	Not tested

## Abbreviations

HAT	Histone acetyltransferases
HDAC	Histone deacetylases
IL	Interleukin
TNF	Tumor necrosis family
LPS	Lipopolysaccharide
IFN	Interferon
MMP	Matrix metalloproteases
PTCL	Peripheral T-cell lymphoma
CTCL	Cutaneous T-cell lymphoma
ART	Antiretroviral therapy
HBV	Hepatitis B virus
HCMV	Human cytomegalovirus
IAV	Influenza A virus
HFpEF	Heart failure with preserved ejection fractions
RA FLSs	Rheumatoid arthritis fibroblast-like synoviocytes
DSS	Dextran sulfate sodium
SLE	Systemic lupus erythematous
RCC	Renal cell carcinoma
